# PDualNet: a deep learning framework for joint prediction of Parkinson’s disease progression subtype and MDS-UPDRS scores

**DOI:** 10.1038/s41598-025-25812-9

**Published:** 2025-11-25

**Authors:** Vasiliki Rizou, Nikos Grammalidis, Petros Daras, Kosmas Dimitropoulos

**Affiliations:** The Visual Computing Lab, Centre for Research and Technology Hellas, Information Technologies Institute, 57001 Thessaloniki, Greece

**Keywords:** Parkinson’s disease, Multi-task learning, Transformer, Progression subtypes, MDS-UPDRS I-III scores, Biomarkers, Computational biology and bioinformatics, Diseases, Neurology, Neuroscience

## Abstract

Parkinson’s disease is one of the most common and complex neurodegenerative diseases, characterized by remarkable motor and cognitive decline. As it is a highly heterogeneous disorder, i.e., the specific symptoms, their severity, and their progression rate manifest significant interpersonal variability, multiple progression subtypes can be defined. The identification and prediction of these subtypes is crucial for understanding the disease’s state and future trajectory, advancing prognostic accuracy and personalized treatment planning. At the same time, the ability to predict future MDS-UPDRS scores, provides an objective assessment of symptoms, supporting clinicians in tracking disease progression and evaluating treatment efficacy. To address both critical objectives, we introduce PDualNet, a novel dual-task framework that jointly models and predicts the disease progression and severity based on longitudinal clinical patient data. Our approach involves two key components: (i) an unsupervised module that maps the single-visit data of each patient, onto a “Single-Visit Embedding (SiVE) space”, and (ii) a supervised part, that utilizes the pre-trained SiVE embeddings to learn a compact representation of the longitudinal data of each patient, representing the “Disease State Embeddings (DiSE)”. These embeddings drive two parallel decoders: one predicting the progression subtype, and the other forecasting the future MDS-UPDRS I–III scores. After analysing patient visit data from up to six years after baseline, each consisting of 89 clinical features, we trained and evaluated PDualNet on 579 participants from the Parkinson’s Progression Markers Initiative. The resulting model, demonstrated remarkable performance on both classification and regression tasks, while additional validation on 490 participants from the Parkinson’s Disease Biomarkers Program cohort, confirmed its robust performance and strong generalization capabilities.

## Introduction

Affecting over 11 million people globally^1^, Parkinson’s disease (PD) is the second most prevalent neurodegenerative disorder^2,3^. According to recent studies^4^ the number of individuals affected is projected to reach 25.2 million by 2050, and in the absence of effective disease-modifying treatments capable of preventing or altering the disease’s progression, PD is expected to impose a considerable burden on public health systems worldwide. The main pathophysiology of the disease includes the progressive degeneration of dopaminergic neurons in the substantia nigra^5,6^, leading to motor symptoms such as bradykinesia, rigidity, resting tremor and gait problems^7,8^. Over time, non-motor symptoms also increase, including, among others, cognitive impairment, sleep disturbances, and mood swings^9,10^. It is worth noting, that PD displays substantial inter-individual variability in symptoms, severity, progression rate, medication responsiveness, and underlying pathophysiology^11^. This inherent and pervasive heterogeneity^12,13^, particularly evident in the early to middle stages poses significant challenges. Major issues include difficulties in designing clinical trials^14^ that accurately assess treatment efficacy, as well as in administering appropriate medication to patients with disease diverse trajectories.

To address these challenges, researchers have prioritized more personalized approaches, focusing on the identification and understanding of the different subtypes of PD progression, as it can help estimate the disease’s course and survival, providing a more accurate prognosis at the time of diagnosis^15^. Although early efforts at subtyping Parkinson’s disease mainly relied on motor symptoms^16^, more recent approaches lean toward integrating both motor and non-motor features^12,17,18^. These newer models are continually being refined through the inclusion of additional data modalities, offering potentially greater clinical utility^19^. Another notable difference is the employment of longitudinal data^20,21^, in place of single time-point predictions, which is crucial for capturing the disease dynamics and progression patterns over time. To achieve this effectively, many research works have extensively utilized Machine Learning (ML) and Artificial Intelligence (AI) methods^22^, for identification and prediction of PD subtypes, monitoring symptoms, and predicting outcomes. In particular, techniques such as unsupervised clustering, and deep learning-based trajectory modelling, offer distinct advantages by modeling nonlinear relationships, handling high-dimensional multimodal datasets, and incorporating longitudinal trajectories, thereby enabling more precise, data-driven stratification of PD patients and paving the way for personalized prognostic and therapeutic strategies.

More specifically, Su Chang et al.^23^ utilized multimodal data from de novo PD patients, to cluster them into three progression subtypes, namely the Inching Pace subtype (PD-I), the Moderate Pace subtype (PD-M) and the Rapid Pace subtype (PD-R). The authors further correlated these subtypes with biomarkers like CSF P-tau/a-synoclein and brain atrophy, while molecular analyses highlighted subtype-specific genes (e.g. STAT3, FYN) and revealed pathways such as neuroinflammation and oxidative stress driving rapid progression. Similarly, A. Dadu et al.^24,25^ performed dimensionality reduction via a non-negative matrix factorization approach along with a Gaussian Mixture Model, to cluster PD participants into the slower-progressors, moderate-progressors and fast-progressors, identifying serum neurofilament light as a significant indicator of fast disease progression among other key biomarkers of interest. For the supervised part, they used simple ML models for early subtype prediction after 60 months, on the three following input cases: (a) from BL clinical factors, (b) from BL and first year (after BL) clinical factors, and (c) from biological and genetics measurements. On the other hand, several works^26,27^ suggest clustering into two subtypes. For instance, Shakya et al.^27^ detected, using a K-Means clustering approach, the Severe Motor-Non-Motor Subtype (SMNS), characterized by older age at onset and more severe motor and non-motor symptoms, and the Mild Motor-Non-Motor Subtype (MMNS), with younger age at onset and milder symptoms. Imaging biomarkers supported these findings, showing greater neural damage in SMNS, while both subtypes suggest significantly different progression patterns in both motor and cognitive functions. Likewise, Hähnel et al.^26^ used a latent time joint mixed-effects model (LTJMM) in addition to a Variational Deep Embedding with Recurrence (VaDER)^28^, to derive two stable PD subtypes (fast and slow progressing) across three large cohorts. For the supervised classification stage, they examined several models including penalized Logistic Regression with L2 regularization, Random Forest, and eXtreme Gradient Boosting (XGBoost). The authors further demonstrated the cross-subtype differences in motor/non-motor progression, survival, treatment response, imaging and digital gait features. In order to deal with irregular time intervals that are common in longitudinal patient records, Baytas et al.^29^ proposed Time-Aware LSTM (T-LSTM), which was then integrated in an autoencoder to learn a powerful single representation suitable for patient subtyping. Similarly, a state-of-the-art recurrent neural network, namely Gated Recurrent Unit (GRU), was used for handling missing values in multivariate time series data^30^, leading to models achieving state-of-the-art performance for classification tasks.

Beyond identifying PD subtypes, it is also important to consider one of the most prevalent and widely used practices for assessing PD progression; the decline in motor symptoms, determined using the Movement Disorder Society Unified Parkinson’s Disease Rating Scale (MDS-UPDRS)^31^. Developing models capable of reliably predicting future MDS-UPDRS scores, can facilitate personalized treatment planning, enable more precise patient stratification, reduce variability in clinical trial cohorts, and yield valuable insights into disease mechanisms and progression. Building on these metrics, Sotirakis et al.^32^ employed wearable sensors and machine learning (notably Random Forest), to accurately predict MDS-UPDRS-III scores in PD patients over time. The developed model detected motor symptom progression within 15 months, outperforming traditional clinical scales, and proposed a Convolutional Neural Network (CNN) trained on baseline gait data, which accurately forecasts MDS-UPDRS III scores after 3 years, highlighting the potential of ML in assessing PD motor severity and supporting clinical management and decision-making. Tsolakis et al.^33^ developed the Ince-PD model, based on Inception architectures for time-series classification, demonstrating high accuracy in predicting MDS-UPDRS I & II scores using wearable data and surveys. Additionally, a Multi-Layer Perceptron model using temporal data from the AMP-PD dataset outperformed other algorithms in forecasting PD progression, with supervised data formatting significantly improving prediction accuracy, as shown by Chowdhury et al.^34^.

While the aforementioned approaches demonstrate promising results, showcasing that data-driven models can uncover early indicators of different motor and non-motor progression types, to this date, there is no explicit attempt to jointly characterize the individual disease trajectories’ subtype along with the future MDS-UPDRS scores; the two gold standards for assessing PD progression. Knowledge of both is considered crucial for a comprehensive understanding of the patient’s clinical status and disease progression. More specifically, the identification of the progression subtype, derived from diverse clinical features, e.g., demographics, motor assessment, MoCA score, medication, allows for a complete view of the disease’s state and its evolution, while the MDS-UPDRS I-III scores provide a robust, quantitative measure of the patient’s motor and non-motor symptoms, enabling clinicians to monitor disease progression and treatment response over time. To this end, in this paper we propose a novel dual-task framework, which models both progression subtype and severity, capturing their inherent interdependence and supporting a holistic and clinically meaningful assessment of the disease’s trajectory. More precisely, as a first step, using an unsupervised approach, we identify three progression subtypes of PD patients, unveiling rapid, moderate and slow progression pace. These subtype labels are then used to supervise the training of PDualNet; a novel multi-task Transformer-based architecture that integrates subtype classification with the prediction of future MDS-UPDRS I–III scores within a unified modeling framework. With a shared encoder for the two tasks, the model effectively learns a joint representation of each patient’s disease state (“Disease State Embedding”), while capturing the underlying latent dependencies between subtype classification and symptom progression. This ultimately yields to strong predictive performance and generalization capabilities across both objectives. The novelty of this work lies in the following contributions:

- A novel deep learning framework is proposed that jointly predicts each participant’s progression subtype and future MDS-UPDRS I–III scores, based on a modelling approach that effectively transforms longitudinal clinical data from each patient, into meaningful representations, i.e., Disease State Embeddings (DiSE). This single-network architecture further enhances data efficiency and computational scalability, while addressing the dual-task objective. Moreover, based on representation learning, this approach enables generalization through cross-task regularization, capturing temporal dynamics that are relevant to both classification and regression tasks.
- A single - unified DL model is introduced with autoregressive capabilities that allows the prediction of entire future trajectories of MDS-UPDRS I-III scores, irrespective of the number of clinical visits available per patient. The proposed model introduces flexibility in handling variable-length sequences and missing data, as well as the ability to monitor and observe long-term dependencies in disease progression.
- The proposed methodology is validated on the large and most widely used cohort of the Parkinson’s Progression Markers Initiative (PPMI), as well as on the external validation cohort of Parkinson’s Disease Biomarkers Program (PDBP), showcasing its superior performance on both tasks over simpler baseline models and demonstrating strong generalization capabilities.

## Methods

In this study, we propose an end-to-end methodology, starting from data preprocessing and identification of the PD progression subtypes, followed by the design and development of a Deep Learning (DL), Transformer-based framework, that addresses the dual task of predicting the progression subtype of each participant along with the future MDS-UPDRS I - III scores. More specifically, we extract different clinical motor and non-motor longitudinal data from large cohorts of PD participants as well as from Healthy Controls (HCs). The data is preprocessed, including cleaning, outlier detection and removal, normalization of specific features, and imputation of missing values. At the next stage of the proposed method, the progression subtypes are identified. This includes an unsupervised approach, which consists of an LSTM-based autoencoder, resulting in longitudinal data embeddings for each participant. The extracted representations, corresponding to PD patients are then clustered into the different progression subtypes of the PD patients: rapid - moderate - slow, while HCs are seen to form by default a separate cluster. The process continues with the PDualNet model, where we firstly train an Autoencoder for capturing the latent representation of each participant’s single-visit data, *Single-Visit Embedding (SiVE) Space*. The Encoder’s projection is then used as the embedding module for a Transformer encoder^35^, modelling the overall disease state representation of all the provided visit data per patient, *Disease State Embeddings (DiSE)*. This learned representation is eventually fed into two separate decoders; a Subtype Decoder for the classification task and a MDS-UPDRS Decoder for addressing the regression task. The model is trained in a three-phase manner to effectively achieve the joint task objective.

**Fig. 1:**
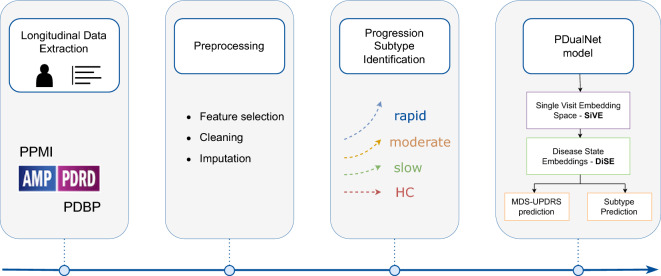
Overview of the proposed methodology: Starting with data extraction and preprocessing, followed by the identification of the PD progression subtypes, and finally the modelling and joint prediction of the Parkinson’s Disease Progression & Severity, using the PDualNet framework.

The overview of the proposed methodology is illustrated in Fig. 1, while a detailed description of each step is provided in the following subsections.

### Preprocessing

The first step consists of the data preprocessing. Initially, for each participant, *p*, its longitudinal data are extracted. This includes demographic information, patient history, and clinical features, such as MDS-UPDRS I-III score, MoCA assessment, UPSIT booklet, Schwab and England ADL (Activities of Daily Living) scale, EPWORTH Sleepiness Scale (ESS), and medication information, analytically described in future sections. These data, are organized in visits $\{BL, V_1, V_2,...,V_n\}$, where *BL* is the Baseline visit and $V_n$ is the last available visit of each patient. The visits are time-ordered, and quantified in 6-month intervals, starting from *BL*. As a result, for each patient, the longitudinal data of each participant *p* are represented as $L_p = \{x_{BL}, x_1, x_2, \dots , x_n\}$, where $x_i$ is the feature vector of the $i^{th}$ visit.

While processing the data, feature selection is simultaneously being performed, as well as partial outlier detection and removal. In terms of the feature selection, there is a notable trade-off between the number of clinically significant features we want to include and the number of participants that have at least a single visit recording per feature. Thus, features that are available in very few participants are excluded from the analysis. Likewise, participants with sparse feature data are removed, thereby maximizing the cohort size with sufficient observations across the selected features. For the resulting sub-set of preserved participants $P = \{p_i\}_{i=1}^{N_p}$ and features $F = \{f_j\}_{j=1}^{N_f}$, the last step consists of data imputation as many participants contain feature vectors with missing values. This is performed using standard imputation techniques, including constant imputation (for static features, e.g. age, sex) and interpolation (for time-varying features, e.g., MDS-UPDRS scores), using least squares regression.

### Progression subtypes extraction

**Fig. 2:**
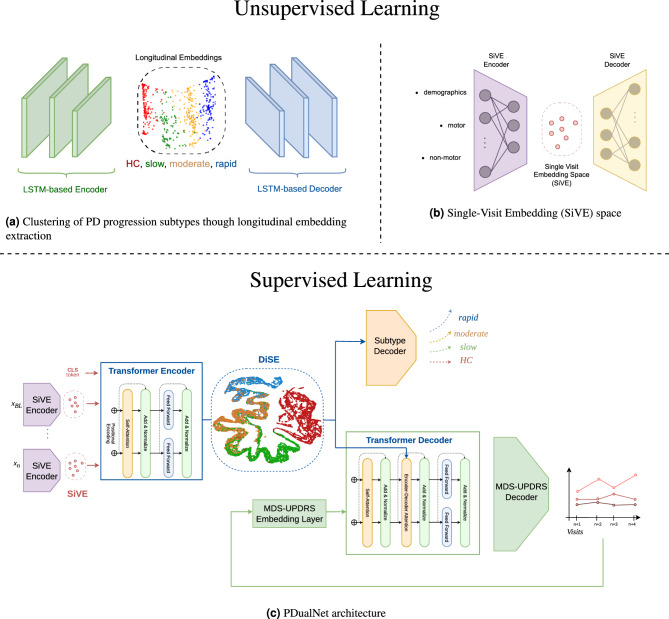
Overview of the subtype extraction and PDualNet architecture. (**a**) Unsupervised module for longitudinal embeddings extraction and clustering of PD progression subtypes into: rapid, moderate and slow. (**b**) Single-Visit Embedding (SiVE) space, where each visit’s feature vector is mapped to a reduced latent representation, using an Autoencoder. (**c**) the SiVE embeddings are used as input to a Transformer Encoder-Decoder based architecture, that models the Disease State of each participant (DiSE), and used this representation to jointly predict the progression subtype and the future MDS-UPDRS I-III scores.

After completing the data preprocessing step, we proceed with the identification of the PD progression subtypes. We first generate a single-vector representation of each participant’s extracted data, irrespective of the number of its available visits. For this purpose, as proposed in similar works^23^ an LSTM-based autoencoder is employed (Fig. 2a), which additionally serves the purpose of dimensionality reduction. The proposed autoencoder is trained using the reconstruction loss in Eq. 1.

1 $$\begin{aligned} \mathcal {L}_{rec}(p) = \left\| L_p - \hat{L_p} \right\| _2^2 = \frac{1}{|L_p|} \sum _{i=BL}^{V_n} \left\| x_i - \hat{x}_i \right\| _2^2, \forall p \in P \end{aligned}$$

Furthermore, the trained autoencoder is utilized for outlier detection and removal. More specifically, we define a threshold $\mathcal {L}_{max}$ for the reconstruction loss. Then for each participant’s *p* longitudinal trajectory, we calculate its reconstruction loss $\mathcal {L}_{rec}(p)$, and if $\mathcal {L}_{rec}(p) > \mathcal {L}_{max}$, *p* is removed from the analysis. The intuition behind this is that the autoencoder learns the underlying distribution of the data points in the dataset, and thus, data points with significant difference from the norm, have a high reconstruction loss, indicating that they are outliers.

Moving forward, by preserving only the encoder part of the trained autoencoder, we extract longitudinal embeddings of each participant. Finally, using the embeddings corresponding to PD patients, we employ unsupervised clustering, namely K-Means, which uncovers the three progression subtypes.

### PDualNet model

The extracted progression subtypes are used as labels for the next phase of the methodology, which is the design and training of the model. PDualNet consists of the following core components:

- *SiVE Autoencoder* This network, illustrated in Fig. 2b, aims to capture the Single-Visit Embeddings (SiVE), $z_i$, of each visit’s $x_i$ feature vector. It is constructed using a Multi-Layer Perceptron (MLP) encoder and decoder. The latter is omitted after pre-training, whereas the former serves as the embedding module for the Transformer encoder. The rationale behind the use of this pre-trained module, as emerged from similar works^36^, lies on providing rich and meaningful initial representation of each visit’s data. Another significant advantage that comes with this modelling approach is the improved performance in terms of stability and training convergence^37^, especially when the available data is limited.
- *DiSE Transformer-Encoder* A Transformer-based encoder that transforms longitudinal clinical data into informative unique Disease-State embeddings (DiSE). Using representation learning, this module contributes to generalization between tasks through shared temporal dynamics, allowing both classification and regression to be beneficial. The input to this module is the sequence of time-ordered SiVE embeddings for a single patient along with a CLS token at the beginning. The latter, is a special token^38^, often used in Transformer-based architectures, placed always at the beginning of the input and is responsible for capturing the representation of the input sequence. The resulting embedding vector $d_p$ for each participant *p* is then provided as input to the two subsequent decoders.
- *Subtype decoder*: A MLP decoder that generates an unnormalized logits vector $s = [s_r, s_m, s_s, s_{HC}]$, assigning a single logit for each one of the classes (C): rapid, moderate, slow and HC respectively. The $\displaystyle \arg \max _j s$ represents the predicted progression subtype of each participant, given their DiSE representation.
- *MDS-UPDRS decoder* A framework consisting of a Transformer decoder, followed by a MLP is responsible for forecasting the future MDS-UPDRS I - III scores of each participant, given their DiSE representation and the previously computed MDS-UPDRS scores. It operates in an autoregressive manner: the MLP’s output, namely, a 3-d vector $u = [u_I, u_{II}, u_{III}]$ of the predicted scores from the previous time-step, is passed through an embedding module and then used as input to the Transformer decoder, together with the DiSE representation.

The detailed architecture of PDualNet is illustrated in Fig. 2c

### Training of PDualNet

**Fig. 3:**
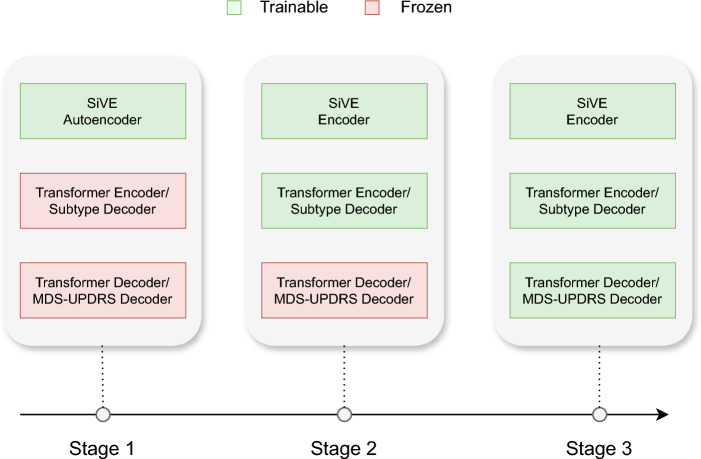
Training phases of PDualNet. The first phase consists of training the SiVE Autoencoder, the second phase is the training of the DiSE Transformer Encoder, and the third phase is the joint training of both decoders.

The final step is the training of the PDualNet network. Due to the model’s complexity and multi-objective training goal, the training is performed in three distinct phases, as depicted in Fig. 3. In the first phase, we train solely the SiVE Autoencoder, to learn the reduced latent representations $z_i$ of each visit $x_i$. This is achieved by minimizing the reconstruction loss in eq. 2.

2 $$\begin{aligned} \mathcal {L}_{rec} = \sum _{p \in P} \sum _{i = 1}^{|L_p|} \left\| x_i - \hat{x_i} \right\| _2^2 \end{aligned}$$

Once, this first phase is complete, we preserve its encoder part and use it as the Transformer Encoder’s embedding module. In the second phase, we train the DiSE Transformer Encoder, with the single objective of subtype classification (i.e. the upper branch of PDualNet in Fig. 2c), while the lower branch of the model, consisting of the MDS-UPDRS Decoder remains frozen. For this part, we minimize the weighted cross-entropy loss $\mathcal {L}_{subtype}$ in Eq. 3, where *s* is the vector of the predicted unnormalized logits, *y* is the ground truth subtype label, provided in the form of class indices in the range {0, ..., C}, *C* is the number of classes, and *B* is the batch size. To mitigate class imbalance and facilitate the convergence during the model’s training, we add class weights, *w*, in the subtype classification loss, ensuring that underrepresented or easily learned subtypes contribute proportionally during optimization.

3 $$\begin{aligned} \begin{aligned} l(s, y)&= \{l_1, l_2, \dots , l_B\}^T, \quad l_n = -w_{y_n} \log \frac{\exp (s_{n, y_n})}{\sum _{c=1}^{C} \exp (s_{n, c})} \\ \mathcal {L}_{\text {subtype}}&= \frac{\sum _{n = 1}^{B}l_n}{\sum _{n=1}^{B} w_{y_n}} \end{aligned} \end{aligned}$$

The last training phase, also involves the MDS-UPDRS Decoder. The loss function consists of two components, namely, the cross-entropy loss as introduced above along with the MSE loss for the regression task, defined in eq. 4, below:

4 $$\begin{aligned} \mathcal {L}_{joint} = {\left\{ \begin{array}{ll} \mathcal {L}_{MSE} & \text {if } \text {epoch} \le \mathcal {E}_{thres} \\ \frac{e^{-w_1}}{2}\mathcal {L}_{MSE} + \frac{e^{-w_2}}{2}\mathcal {L}_{\text {subtype}} & \text {if } \text {epoch} > \mathcal {E}_{thres} \end{array}\right. } \end{aligned}$$

where $\mathcal {L}_{MSE} = \alpha _1 \left\| u_I - \hat{u}_I \right\| _2^2 + \alpha _2 \left\| u_{II} - \hat{u}_{II} \right\| _2^2 + \alpha _3 \left\| u_{III} - \hat{u}_{III} \right\| _2^2$, with $\hat{u}_I, \hat{u}_{II}, \hat{u}_{III}$ being the predicted MDS-UPDRS I, II and III scores respectively, and $\alpha _1, \alpha _2, \alpha _3$ being the weights for each score. The weights $w_1$ and $w_2$ are used to balance the two components of the loss function. The exponential term is used for stability, when the weights are trainable parameters. Lastly, the threshold $\mathcal {E}_{thres}$ acts as a “warm-up” phase, where the upper branch (i.e. SiVE Encoder, DiSE Transformer Encoder, and Subtype Decoder) of the model is frozen for same epochs, allowing the model to have a better initialization before the joint training phase. A point worth mentioning is that, since the MDS-UPDRS Decoder operates in an autoregressive manner, we use *teacher forcing*^39^ during training (i.e. the ground-truth MDS-UPDRS scores, $u_I[t], u_{II}[t], u_{III}[t]$ are fed as input at each time step $t + 1$). In contrast, during inference, the model uses its own previously predicted scores $\hat{u}_I[t], \hat{u}_{II}[t], \hat{u}_{III}[t]$ to forecast the next time step.

Lastly, to fully leverage the model’s capabilities, we perform a final data preparation step. More concretely, we split the dataset (i.e. the PPMI cohort) into a training and test set, containing $q \%$ and $(100 - q) \%$ of the participants in the cohort respectively, to maintain consistent, non-overlapping samples for all training phases. Afterwards, using a sliding window approach, we create multiple, highly correlated training samples, as follows: for each participant’s longitudinal data $L_p = \{x_{BL}, x_1, x_2, \dots , x_n\}$, given a window size $W < |L_p|$, this yields $|L_p| - W$ overlapping input sequences (i.e.$\{x_{BL}, x_1, \dots , x_{W - 1}\}$, $\{x_{1}, x_2, \dots , x_{W}\}$, ..., $\{x_{|L_p| - W + 1}, x_{|L_p| - W + 2}, \dots , x_{n - 1}\}$). Additionally, given that the model is designed to predict multiple future MDS-UPDRS scores, for each input sequence, e.g. $\{x_i, \dots , x_{i + W - 1}\}$, there could be up to $|L_p| - W - i$ different training samples, each corresponding to a different number of MDS-UPDRS scores to be predicted. It can be seen that there are $N(p, W) = (|L_p| - W)(|L_p| - W + 1) / 2$, possible training samples per participant *p* and sliding window size *W*. The same procedure is applied to the test set as well. This acts not only as a form of data augmentation, increasing significantly the number of train and test instances, but also improves the model’s ability to generalize across varying input lengths and forecast horizons.

## Datasets

In this work, two datasets were considered, the Parkinson’s Progression Markers Initiative (PPMI)^40^ used for both training and validation and the Parkinson’s Disease Biomarkers Program (PDBP)^41^ used as external validation for the proposed methodology, both extracted from the AMP-PDRD Knowledge Platform.

**Fig. 4:**
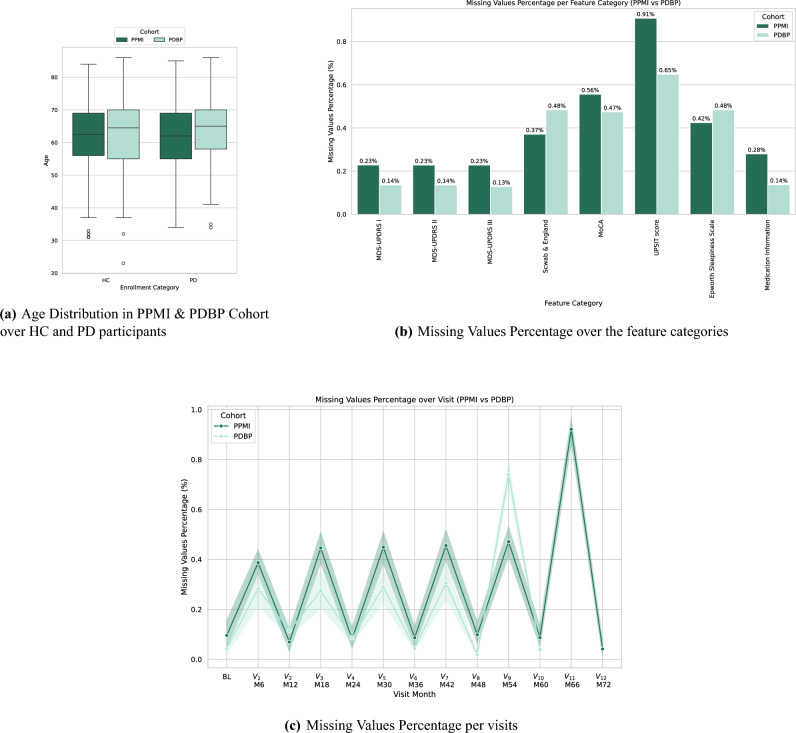
Summary of the utilized datasets. (**a**) Age distribution in both cohorts, over HC and PD participants. (**b**) Missing values over the extracted feature categories. (**c**) Total missing values percentage over the available visits in each cohort.

PPMI is a large, well-characterized cohort of thousands of individuals, with and without Parkinson’s Disease, aiming to identify critically needed biological markers of Parkinson’s onset and progression. In our analysis, after preprocessing, we considered 579 participants, comprising of (N= 391) PD patients (34% Male - 66% Female) and (N= 188) healthy controls (HC)(35.6% Male - 64.4%Female). We extracted their longitudinal data, accounting for a total of 89 clinical features, across the following categories:

- *Demographics* Including age at BL, sex, race and years of education.
- *Patient History* Including if the participant has a family member (e.g. parent or other relative) with PD.
- *MDS-UPDRS I* The MDS-UPDRS Part I score, consisting of 13 features, assessing non-motor aspects of daily living.
- *MDS-UPDRS II* The MDS-UPDRS Part II score, including 13 features, considering motor aspects of experiences in daily living.
- *MDS-UPDRS III* The MDS-UPDRS Part III score, containing 18 distinct features, which is the clinically assessed motor examination.
- *Schwab and England ADL* The Schwab and England ALD score^42^, which is a 100-point scale that addresses the capabilities of people with impaired mobility.
- *MoCA* The Montreal Cognitive Assessment score^43^, which is designed as a rapid screening instrument for mild cognitive dysfunction. We utilize five relevant features, plus the total score.
- *UPSIT* The University of Pennsylvania Smell Identification Test (UPSIT) booklet^44^, including 4 features (i.e. 4 different 10-page booklets), responsible for assessing the olfactory function of the participants.
- *ESS* The Epworth Sleepiness Scale^45^, including eight features, responsible for assessing a person’s average sleep propensity, or how likely they are to fall asleep in various situations
- *Medication* Includes three features, namely, whether the participants is on Levodopa, Dopamine Agonist or other PD medication.

The number of visits taken into account for the case of the PPMI cohort, ranges from 1 (BL) to 13 ($V_{12}$), positioned at fixed 6-month intervals. Intermediate 3-month visits were excluded, due to a higher proportion of missing values and their absence in the PDBP cohort.

On the other hand, regarding the PDBP cohort, we preserved 490 participants, consisting of (N= 280) PD patients (57.9 % Male - 42.1 % Female) and (N= 210) healthy controls (HC) (46.7 % Male - 53.3 %). For comparison purposes, the feature set was kept consistent with the PPMI cohort (89 features), and the number of available extracted visits ranges from 1 (BL) to 11 ($V_{10}$), all spaced at 6-month intervals.

Additional statistical information regarding the two cohorts is provided in Fig. 4. In particular, Fig. 4a

illustrates the age distribution of the participants in PPMI and PDBP, across the PD and HC groups. On the other hand, Fig. 4b

provides a missing values percentage distribution over the discrete feature categories, while Fig. 4c corresponds to the missing values percentage, over all features and participants, per visit. It can be observed that the PPMI cohort exhibits a higher percentage in missing values in most feature categories, while both cohorts show a similar trend in the missing values per visit, with yearly visits, i.e., $BL, V_2, V_4$, having a decreased rate of absent values, compared to the intermediate visits, i.e., $V_1, V_3,$ and so forth.

## Method evaluation

This section demonstrates the effectiveness of the proposed methodology by presenting the imputation and clustering results and evaluating the performance of the PDualNet model. The model is assessed across both the PPMI and PDBP cohorts for each of the two tasks. Additionally, we compare the performance of PDualNet with three baseline models to highlight its advantages.

### Imputation

For handling missing values, our approach combines constant imputation and linear regression, offering a balance between interpretability, computational efficiency, and temporal consistency. To evaluate our choice, we further conducted an analysis comparing the original imputation pipeline with two widely used alternative strategies, i.e. KNN imputation^46^ and Multivariate Imputation by Chained Equations (MICE)^47^. More specifically, we compared the mean and std. deviation of each feature, before and after each imputation technique was applied and report their respective differences in Table 1. Although all methods appear to preserve the initial feature distributions, we utilized the aforementioned imputation approach, as it achieves the lowest deviation across all three methods.

**Table 1:** Comparison of imputation strategies across PD and HC cohorts in PPMI dataset. Each value represents the mean absolute distributional shift between the original (non-imputed) and imputed feature distributions, computed for the mean, standard deviation, and average z-score distance. Lower values indicate smaller distortion of the underlying data statistics.

	PD cohort			HC cohort		
abs. dist. shift	Ours	kNN	MICE	Ours	kNN	MICE
Mean	**0.011**	0.013	0.025	**0.012**	0.018	0.026
Std	**0.013**	0.023	0.016	**0.032**	0.040	0.041
Z-score	**0.010**	0.014	0.024	**0.022**	0.039	0.052

Significant values are in bold.

### Clustering

The clustering procedure is performed on the PPMI cohort and the trained model (i.e. LSTM Autoencoder) as well as the determined cluster centroids are used to extract the labels for the PDBP cohort, as it represents our external validation set.

The LSTM Autoencoder, consisting of a single Layer in both the encoder and decoder part, with a hidden dimension (i.e., the embedding dimension) of 32, is trained for (N= 70) epochs, using the Adam optimizer with a learning rate of $2 \times 10^{-3}$.

Upon completion of the training, K-Means clustering is performed for extracting the progression subtypes. For determining the number of clusters *k*, we examined the alternatives $k=2$ and $k=3$, as they represent the common practice in the latest literature^23,26^. We assessed their respective clustering performance using the silhouette score, the Davies-Bouldin index, and the Akaike Information Criterion (AIC), as summarized in Table 2. Since $k=3$ produced the best results in two of the three cases and is also the most prevalent choice in related studies, we adopted $k=3$ for subsequent analysis.

**Table 2:** Comparison of AIC, Silhouette score and Davies-Bouldin index for the two dominant choices ($k=2$ and $k=3$) for the number of progression subtypes.

N clusters	AIC ($\downarrow$)	Silhouette ($\uparrow$)	Davies-Bouldin index ($\downarrow$)
$k = 2$	− 111698.99	**0.294**	1.43
$k = 3$	**− 115456.17**	0.288	**1.36**

Significant values are in bold.

Thus, three clusters were formed, representing the distinct progression subtypes, i.e. rapid (with the faster progression pace), moderate (with an intermediate pace) and slow (with the milder symptom manifestation).

**Fig. 5:**
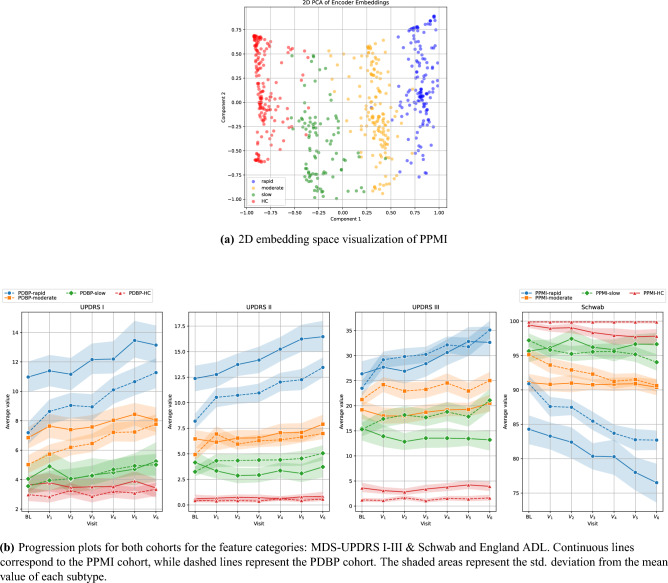
Visualization of the clustering analysis results. (**a**) The projected 2D embedding space of the longitudinal latent representations of the participants in PPMI. The three clusters appear coloured in blue, yellow and green, representing the rapid, moderate and slow progression subtypes, respectively. For the sake of completeness, the HC participants are included, in red colour. (**b**) The progression plots of the three subtypes, in the PPMI cohort (continuous lines) and the PDBP cohort (dashed lines). The shaded areas represent the std. deviation from the mean value of each subtype.

Specifically, for the PPMI cohort, the clustering unveiled (N= 145) rapid, (N= 152) moderate and (N=94) slow progression patients. The clustering result can be found in Fig. 5a, illustrating the 3 clusters (plus the HC participants). For visualization purposes, the projected 2D longitudinal embedding space is derived using Principal Component Analysis (PCA)^48^ on the latent representations of the participants. When it comes to the PDBP cohort, using the trained LSTM encoder to derive the latent representation embeddings, followed by the prediction of the cluster labels, based on the determined centroids, we detected (N= 112) rapid, (N= 128) moderate and (N = 40) slow PD patients. We additionally provide the mean distribution of certain feature categories, including the MDS-UPDRS I-III scores along with the Schwab and England ADL scores, which are of particular importance for our analysis, in Fig. 5b. Although the two cohorts exhibit differences - as expected, due to the heterogeneity of the disease and patients in each group - the progression subtypes appear to be consistent across the two.

### PDualNet training and evaluation

#### Data preparation and baselines

For the training of PDualNet we split the cohort into training and test set, containing 75% and 25% of the participants respectively, stratified with respect to the progression subtypes (109/36 for rapid, 114/38 for moderate, 70/24 for slow and 141/47 for HC). Additionally, we further split the training set into 5 stratified subsets, to perform a 5-fold cross validation. To generate the input sequences, we use a sliding window for each participant, with sizes $W = \{2, 3, 4, 5\}$, and for training we set the maximum number of future MDS-UPDRS scores to predict to 5.

For performance comparison, we use the same data split to train and test three baseline models. The first is a Temporal Convolutional Neural Network (TCN)^49^ -based model, consisting of a TCN encoder followed by two Multilayer Perceptron (MLP) decoders-one for classification and one for regression. Our second baseline shares the same encoder-decoder architecture, but with the encoder replaced by a Long Short-Term Memory (LSTM) module. Both aforementioned models were trained following a two-phase procedure analogous to that of our proposed model, ensuring clarity and fair comparison. Specifically, in the first phase, the models were trained on the classification task alone, with the regression decoders’ parameters kept frozen; in the second phase, both tasks were jointly optimized using the dual-task objective. The third baseline comprises two separate Random Forest (RF) models, each dedicated to classification and regression, respectively. However, since these baseline models do not support multi-length input/output sequences, we train different models for each sliding window size *W*, and set the number of future MDS-UPDRS scores to one, to ensure a fair comparison. Testing of all models (including PDualNet) is similarly conducted separately for each *W*, predicting a single future MDS-UPDRS I-III score.

#### Training and implementation details

The number of layers, hidden dimensions, and other hyperparameters of the PDualNet are summarized in Table 3, accounting for a total of 34,703 trainable parameters. The model is trained with batch sizes of 8, 64, 128 and a learning rates of $10^{-3}$, $3 \times 10^{-4}$ and $3 \times 10^{-4}$ for each one of the three training phases, in that order. Regarding the baselines, the TCN-based model, consists of a two-layer TCN encoder with kernel size 2, dilation factors of 1 and 2, and a dropout rate of 0.2. It operates over 89 input channels (one for each input feature) and produces 16 latent feature maps per layer. The two task-specific decoder heads contain a single hidden layer of 16 units with ReLU activation and dropout of 0.1, followed by output layers of dimensions 3 and 4, respectively. In total, the model includes 6.615 trainable parameters. Regarding the LSTM-based model, its encoder contains a single LSTM layer with a hidden dimension of (N= 16) neurons, followed by two MLP decoders, identical to those used in the TCN model, yielding a total of 7511 trainable parameters. Both models are trained with batch sizes of 64 and 128 and a learning rate of $5 \times 10^{-4}$ across the two different training phases. The RF models are trained with 50 and 300 estimators for the classification and regression tasks, respectively.

All experiments were conducted on a Linux server (AMD Ryzen 9 5950X CPU, 64GB RAM) with two Nvidia 3090Ti GPUs (24GB). The PDualNet training, including all three phases, was completed in approximately 4h, while the average inference time of a single test instance is 0.0062s.

**Table 3:** PDualNet detailed structure and parameters.

Component	Structure/Layers	Dropout
SiVE autoencoder	FC layers: $89 \rightarrow 128 \rightarrow 64 \rightarrow 32 \rightarrow 16$	0.3
DiSE transformer-encoder	Positional Encoding 1 Transformer Encoder Layer 1 head Feed-forward dimension: 32	0.2
Subtype decoder	FC layers: $16 \rightarrow 8 \rightarrow 4$	–
MDS-UPDRS decoder	Positional Encoding 1 Transformer Decoder Layer 1 head Feed-forward dimension: 32 FC layers: $16 \rightarrow 16 \rightarrow 3$	0.1
Decoder embedding	3 layers - one for each score, Vocabulary size: 101 Padding index: 100 Projection dimension: 16	–

#### Testing on PPMI cohort

**Fig. 6:**
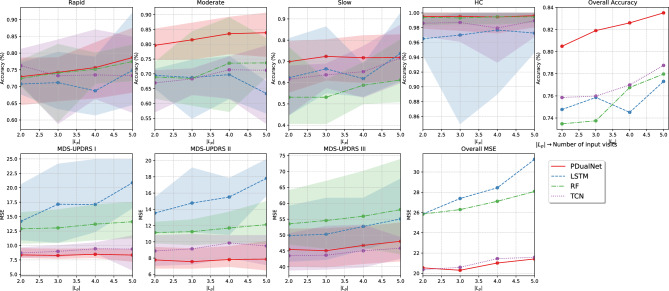
5-fold cross-validation results on the PPMI cohort.

We begin by evaluating the model’s performance on the PPMI cohort, using a 5-fold cross-validation approach, followed by testing the held-out test set. Figure 6 illustrates the cross-validation results, where the solid lines represent the mean performance for each model, while the shaded regions denote the range between minimum and maximum values across varying numbers of input visits. Notably, PDualNet consistently outperforms the baseline models in both tasks and exhibits a more stable performance across the different input sequence lengths. Furthermore, it exhibits low variability around the mean, indicating greater robustness to different training and testing conditions.

For the held-out test set, we present the mean and standard deviation results for varying numbers of input visits (ranging from 2 to 5), across several evaluation metrics, which are summarized in Table 4. More specifically, considering the subtype classification task (Table 4a), PDualNet surpasses both baseline models, achieving the highest overall accuracy (82.87 ± 1.06), precision (83.48 ± 1.18), recall (81.68 ± 1.16) and F1 score (82.37 ± 1.16), retaining at the same time minimal standard deviation, indicating a stable and consistent performance. Another point worth mentioning is that PDualNet is the only model that achieves perfect accuracy for the HC participants, while it performs reliably across all PD subtypes. Lastly, since the task must meet rigorous performance standards, we underline the model’s ability to retain high precision, adding to model’s safety, reliability and effectiveness.

When it comes to the regression learning objective (Table 4b), our model demonstrates the lowest MSE (19.62 ± 0.19), RMSE (4.11 ± 0.01) and highest $R^2$ (0.81 ± 0.01) for the MDS-UPDRS I-III scores. Seemingly, neither of the LSTM and $RF_{reg}$ models can reliably predict all three sub-scores, while all baselines are constrained to a single-step prediction horizon, further highlighting the advantage of PDualNet’s more comprehensive and robust modeling capability.

When it comes to the model’s autoregressive capabilities, we evaluate the model’s performance on several future prediction horizons. The results, reported in Table 4c, indicate stable prediction performance, with controlled and gradual error increase for longer prediction horizons. Specifically, the mean RMSE rises from 4.72 (p.h. = 2) to 5.15 (p.h. = 4), indicating moderate degradation in multi-step forecasting accuracy. The RMSE values by prediction horizon exhibit similar trends, confirming that the PDualNet maintains consistent predictive behavior rather than experiencing abrupt degradation at any specific horizon.

To assess the statistical significance of the discussed performance differences between PDualNet and the baselines, we conducted paired Wilcoxon signed-rank tests, applied separately for both regression and classification metrics. For classification, we computed the error as $1-p$, where p denotes the probability assigned by the model to the true class *c*. The results, present in Table 4d, indicate statistically significant improvements with all *p*-values below 0.05 in all comparisons.

**Table 4:** Performance comparison of models across all input sequence lengths (mean ± standard deviation) - PPMI.

Model	Rapid	Moderate	Slow	HC	Overall	Precision	Recall	F1 Score
	$Acc\, (\%)$	$Acc\, (\%)$	$Acc\, (\%)$	$Acc\, (\%)$	$Acc\, (\%)$			
(a) Subtype prediction performance								
*PDualNet*	78.87 ± 2.26	76.42 ± 1.57	71.43 ± 1.08	100 ± 0.00	**82.87 **±** 1.06**	**83.48 **± ** 1.18**	**81.68 **±** 1.16**	**82.37 **±** 1.16**
*TCN*	80.50 ± 1.7	71.54 ± 5.12	73.77 ± 1.3	97.89 ± 0.61	81.72 ± 0.89	82.01 ± 1.20	80.92 ± 0.80	81.31 ± 1.00
*LSTM*	74.97 ± 5.27	73.09 ± 3.30	69.16 ± 8.94	97.99 ± 2.05	79.93 ± 1.56	79.98 ± 1.68	78.80 ± 1.73	79.14 ± 1.62
$RF_{class}$	80.15 ± 1.80	70.5 ± 4.58	60.32 ± 4.42	98.68 ± 0.42	79.21 ± 2.20	78.65 ± 2.22	77.41 ± 2.72	77.82 ± 2.40

Model	MDS-UPDRS I	MDS-UPDRS II	MDS-UPDRS III	Overall *MSE*	Overall *RMSE*	Overall $R^2$		
	*MSE*	*MSE*	*MSE*					
(b) MDS-UPDRS I-III score prediction performance								
*PDualNet*	9.15 ± 0.18	8.30 ± 0.10	41.39 ± 0.74	**19.62 **±** 0.19**	**4.11 **±** 0.01**	**0.81 **±** 0.01**		
*TCN*	9.29 ± 0.57	10.32 ± 1.01	41.32 ± 1.22	20.31 ± 0.51	4.23 ± 0.07	0.80 ± 0.01		
*LSTM*	16.41 ± 4.61	16.99 ± 3.31	45.31 ± 1.42	26.24 ± 2.68	4.95 ± 0.31	0.69 ± 0.05		
$RF_{reg}$	16.01 ± 0.90	14.89 ± 0.64	50.02 ± 1.45	26.97 ± 0.64	4.98 ± 0.07	0.70 ± 0.00		

p.h.	Mean RMSE				RMSE by Prediction Horizon			
	MDS-UPDRS I	MDS-UPDRS II	MDS-UPDRS III	Overall	MDS-UPDRS I	MDS-UPDRS II	MDS-UPDRS III	Overall
(c) Autoregressive MDS-UPDRS I–III Score Prediction Performance. Mean RMSE values summarize the mean prediction errors up to each prediction horizon (p.h.), while RMSE by p.h. represents the prediction error specifically at each horizon								
2	3.20	3.11	6.85	4.72	3.35	3.34	7.16	4.96
3	3.35	3.32	7.14	4.94	3.66	3.74	7.69	5.37
4	3.54	3.56	7.37	5.15	4.03	4.27	8.14	5.79

Comparison	Task	Statistic	*p*-value					
(d) Wilcoxon signed-rank test for statistical comparison of PDualNet against baselines for regression and classification tasks. Extremely small *p*-values are reported as $p < 10^{-15}$								
*PDualNet* vs. *LSTM*	Regression	1.937.608,5	$p < 10^{-15}$					
*PDualNet* vs. *LSTM*	Classification	1.641.988,0	$p < 10^{-15}$					
*PDualNet* vs. $RF_{reg}$	Regression	1.852.861,5	$p < 10^{-15}$					
*PDualNet* vs. $RF_{class}$	Classification	2.002.570,5	$p < 10^{-15}$					
*PDualNet* vs. *TCN*	Regression	18.983.508,5	$6.27\times 10^{-12}$					
*PDualNet* vs. *TCN*	Classification	1.349.853.0	$p < 10^{-15}$					

Significant values are in bold.

### External validation on PDBP

To put into the test the generalization capabilities of PDualNet, we evaluate its performance on the PDBP cohort. No further fine-tuning is applied to any of the models. The cluster labels derived in the earlier clustering step are used as ground truth, and the results are summarized in Table 5. To provide additional clarity and detail, we analytically present the classification report and regression performance in the form of confusion matrix and actual versus predicted scatter plots in Fig. 8, for the input sequence length of 2 visits (i.e., BL + 1 visit follow-up). Similar performance was observed for the other input sequence lengths, but we focus on the two-visit case for brevity and as this is the most challenging scenario, given the least amount of information available.

PDualNet maintains strong performance across both objectives, achieving an overall accuracy of 82.13 ± 0.80, precision of 78.70 ± 1.38, recall of 79.22 ± 1.17 and F1 score of 78.89 ± 1.31 for the subtype prediction task. Looking into the model’s regression performance, it achieves an overall MSE of 20.44 ± 0.96, RMSE of 4.28 ± 0.11 and $R^2$ of 0.78 ± 0.00 for the MDS-UPDRS I-III scores. On the other hand, its autoregressive behavior further indicates that the model effectively captures longitudinal progression patterns while preserving reasonable accuracy for short- to mid-term forecasts. The aforementioned results closely align with those achieved in the internal test set of the PPMI cohort, showcasing PDualNet’s robustness and generalization capabilities across different datasets and varied input sequence lengths. Another observation is that the baseline models present biases towards certain subtypes. For instance, the $RF_{clas}$ demonstrates a clear preference for the moderate subtypes, even in the PPMI test set, while producing erratic predictions for the remaining subtypes. When it comes to the LSTM model, it achieves significantly higher accuracy for the slow subtype, while the other two subtypes are predicted less reliably - a pattern not observed when tested on the PPMI data, suggesting the model’s difficulty to generalize across cohorts. Similar observations can be made for the TCN model as well, as a bias is observed toward predicting the rapid progression subtypes, with great variance in the model’s performance metrics for the moderate and slow subtype, compared to the PPMI case. These classification trends among the different subtypes for the two baseline models are also illustrated in the confusion matrices of Fig. 8. In contrast, PDualNet shows no such biases, and exhibits stable cross-cohort performance, further demonstrating its robustness.

**Fig. 7:**
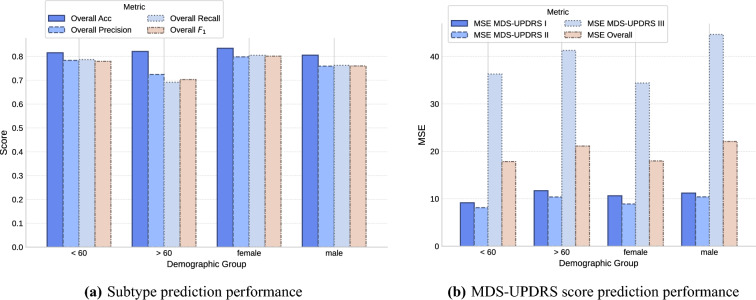
Stratified analysis of PDualNet performance over different Demographic groups, including female, male, participants under 60 years (< 60) and those over 60 years (> 60). Similar performance is observed over all subgroups, indicating no significant algorithmic bias.

To gain greater insight into the model’s generalization capabilities, we perform a stratified analysis across different demographic subgroups of the PDBP cohort. Specifically, we assessed PDualNet’s performance separately for male and female participants, as well as for younger (<60 years) and older ($\ge$ 60 years) individuals, in both the classification and regression tasks. As illustrated in Fig. 7, across all subgroups, classification accuracy ranged from 0.805 to 0.834 and $F_1$-score from 0.703 to 0.801, indicating consistent model performance, and suggesting fairness and external generalization. Minor reductions, within accepted ranges, are observed in recall and MSE in older and male participants, which may reflect sample distribution or clinical heterogeneity rather than algorithmic bias.

Similarly to the PPMI case, we also provide the paired Wilcoxon signed-rank test results. The test statistics are consistently large in magnitude, and all corresponding p-values fall well below conventional significance thresholds (*p* < 0.005), indicating that the observed performance gains are highly unlikely to have occurred by chance. Even in the least significant comparison (regression PDualNet vs. TCN, were *p* = 0.0023 < 0.005), PDualNet still shows a statistically robust improvement.

**Table 5:** Performance comparison - PDBP.

Model	Rapid	Moderate	Slow	HC	Overall	Precision	Recall	F1 Score
	$Acc\, (\%)$	$Acc\, (\%)$	$Acc\, (\%)$	$Acc (\%)$	$Acc\, (\%)$			
(a) Subtype Prediction Performance								
*PDualNet*	77.70 ± 2.87	75.21 ± 0.55	68.13 ± 1.50	95.86 ± 0.82	**82.13 **±** 0.80**	**78.70 **±** 1.38**	**79.22 **±** 1.17**	**78.89 **±** 1.31**
*TCN*	78.27 ± 2.01	69.40 ± 4.05	67.26 ± 13.58	91.44 ± 1.17	78.99 ± 1.51	75.57 ± 1.70	76.59 ± 3.63	75.63 ± 2.16
*LSTM*	70.07 ± 3.79	64.39 ± 3.58	79.27 ± 12.70	90.07 ± 1.93	75.69 ± 2.50	72.11 ± 2.86	75.95 ± 3.98	72.68 ± 3.02
$RF_{class}$	70.06 ± 2.58	78.76 ± 2.51	62.01 ± 4.81	96.30 ± 1.39	80.66 ± 0.77	77.53 ± 1.48	76.78 ± 1.76	76.86 ± 1.70

Model	MDS-UPDRS I	MDS-UPDRS II	MDS-UPDRS III	Overall *MSE*	Overall *RMSE*	Overall $R^2$		
	*MSE*	*MSE*	*MSE*					
(b) MDS-UPDRS I-III Score Prediction Performance								
*PDualNet*	11.19 ± 0.50	9.96 ± 0.85	40.17 ± 1.60	**20.44 **±** 0.96**	**4.28 **±** 0.11**	**0.78 **±** 0.00**		
*TCN*	11.17 ± 0.51	11.61 ± 1.05	38.77 ± 1.01	20.51 ± 0.57	4.42 ± 0.08	0.77 ± 0.01		
*LSTM*	16.98 ± 4.44	19.13 ± 4.26	44.86 ± 3.60	26.99 ± 3.95	5.04 ± 0.40	0.67 ± 0.05		
$RF_{reg}$	15.60 ± 0.94	17.89 ± 1.56	49.62 ± 0.71	27.70 ± 0.96	5.07 ± 0.11	0.68 ± 0.01		

p.h.	Mean RMSE				RMSE by Prediction Horizon			
	MDS-UPDRS I	MDS-UPDRS II	MDS-UPDRS III	Overall	MDS-UPDRS I	MDS-UPDRS II	MDS-UPDRS III	Overall
(c) Autoregressive MDS-UPDRS I–III Score Prediction Performance. Mean RMSE values summarize the mean prediction errors up to each prediction horizon (p.h.), while RMSE by p.h. represents the prediction error specifically at each horizon								
2	3.44	3.34	6.68	4.75	3.55	3.55	6.97	4.96
3	3.56	3.57	6.96	4.96	3.80	4.02	7.47	5.37
4	3.70	3.78	7.21	5.16	4.10	4.42	7.96	5.77

Comparison	Task	Statistic	*p*-value					
(d) Wilcoxon signed-rank test for statistical comparison of PDualNet against baselines for regression and classification tasks. Extremely small *p*-values are reported as $p < 10^{-15}$								
*PDualNet* vs. *LSTM*	Regression	70.466.069	$p < 10^{-15}$					
*PDualNet* vs. *LSTM*	Classification	7.932.208	$p < 10^{-15}$					
*PDualNet* vs. $RF_{reg}$	Regression	68.899.606,5	$p < 10^{-15}$					
*PDualNet* vs. $RF_{class}$	Classification	6.814.771,5	$p < 10^{-15}$					
*PDualNet* vs. *TCN*	Regression	78.284.353	0.0023					
*PDualNet* vs. *TCN*	Classification	7.044.716,5	$p < 10^{-15}$					

Significant values are in bold.

**Fig. 8:**
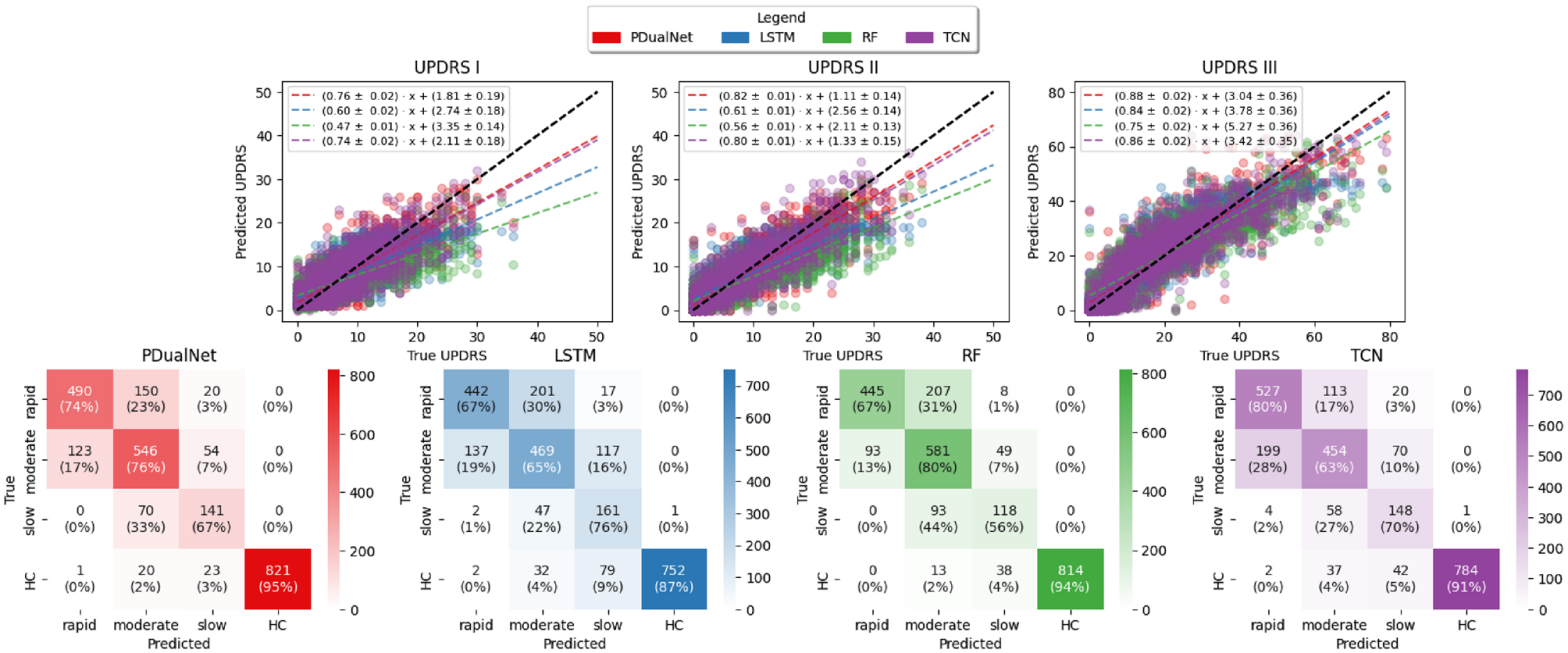
Actual vs Predicted values for the MDS-UPDRS I-III scores for the PDBP cohort for the three models, along with the confusion matrices for the subtype classification for number of input visits equal to 2. Colored dashed lines represent the least-squares fit for each MDS-UPDRS score and model, while the ± values indicate the $95\%$ confidence intervals.

### Model interpretability and forecasting

This section aims to shed light on the *DiSE* embeddings structure, which represent the key component for both learning objectives. In addition, we perform post-hoc explainability analysis using SHapley Additive exPlanations (SHAP)^50^ to extract feature contributions across progression subtypes. Finally, we demonstrate the model’s autoregressive predictive capabilities through representative examples.

**Fig. 9:**
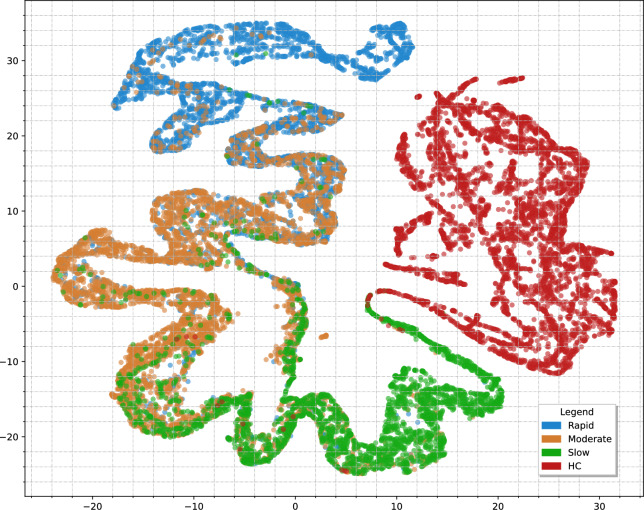
Visualization of disease state embeddings—DiSE space, using a 2D UMAP projection.

Starting with the former aspect, we use a dimensionality reduction technique, namely, UMAP^51^, to project the *DiSE* embeddings into a 2D space, as shown in Fig. 9. The visualization unravels well-separated clusters between the four distinct subtypes. In more detail, the HC group is clearly isolated from the other three PD subtypes, accounting for the perfect accuracy achieved in the classification task. The remaining three classes also form separate regions with smooth transitions between them. Elaborating on the latter observation, the emerging continuity between the subtypes ($slow \rightarrow moderate \rightarrow rapid$), suggests that the model allows for gradual transitions between them. This is a significant remark, as it indicates that the model reflects clinical reality and captures the disease’s dynamic, as the progression subtypes are not rigid, phenotype snapshots, but rather time-informed evolving states.

Moreover, it is also observed that the greatest overlap occurs between the moderate region and its neighbouring subtypes. To some extent, this entanglement is anticipated, if we consider the greater permissive variability of the moderate subtype, compared to the other two. For instance, patients with extreme progression patterns, even when their disease course is substantially distinct from the class mean, are still observed closer to the rapid cluster, analogously to the very slow progression cases. Consistent with this, the embedding reflects patterns observed in the confusion matrix: rapid cases are misclassified only as moderate, and slow cases are similarly confused only with moderate, whereas misclassifications for the moderate subtype are distributed across both slow and rapid classes. Conclusively, although not a direct decision-level attribution, the above analysis suggests that the embedding space provides strong representation-level insights to the model’s decision-making process and underlying learned structure.

**Fig. 10:**
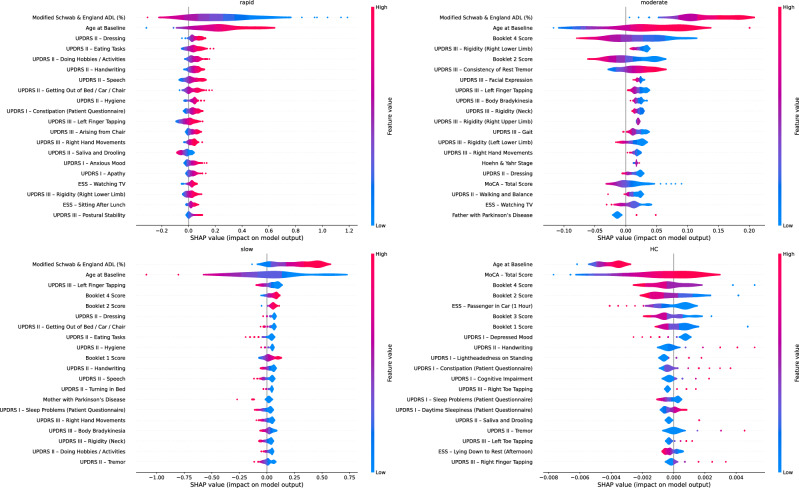
Violin plot—SHAP values.

In order to provide a deeper understanding and transparency regarding the model’s decision-making process, we provide the SHAP values of the most significant features for each subtype. The results are illustrated in the form of a violin plot in Fig. 10, where positive (or negative) SHAP values indicate that the feature pushes the prediction closer to (or further from) the final output. The subplots reveal a clear, clinically interpretable structure in the model’s decision space: high-magnitude motor features, i.e., MDS-UPDRS-III items are the dominant drivers that push predictions toward pathological subtypes, whereas preserved global function and cognition (e.g., higher ADL/Modified Schwab & England, higher MoCA score) tend to push predictions away from rapid decline and toward slower courses or HC. Non-motor features (daytime sleepiness, EPWORTH score items, constipation, mood) contribute more subtly but consistently, enhancing the separation of moderate and slow subtypes from each other and from HC. Moreover, the participant’s age is considered in all subtypes, suggesting that younger patients experience slower disease progression, and vice versa. The color gradients in the summary plots show that higher values of motor-severity features produce positive SHAP contributions for identifying fast progressors, whereas higher cognitive/ADL scores produce negative contributions for those labels, while the exact opposite pattern is observed for the slow subtype. Overall, the SHAP-driven explainability is both informative and clinically meaningful, as it maps the model’s decisions onto familiar, domain-relevant measurements. It should be noted though, that SHAP describes the model’s associations, not causation, and further analysis should be made before clinical deployment.

**Fig. 11:**
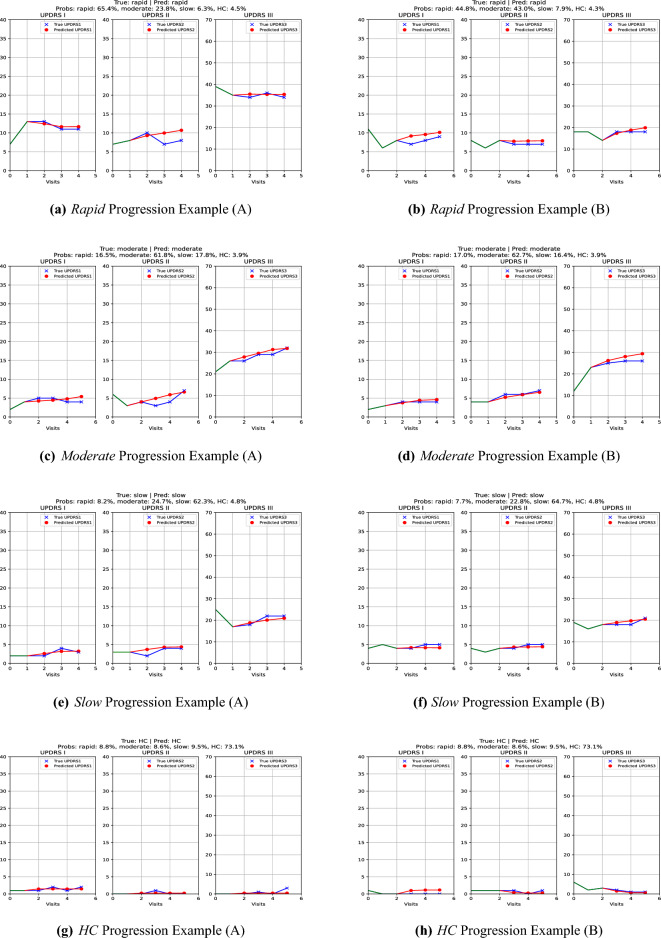
Example of future MDS-UPDRS I-III score predictions for each subtypes. The blue line represents the future score (ground truth), while the red line indicates the model’s predictions. The green part corresponds to the visits used as input to the model.

Since the model is trained to predict multiple future MDS-UPDRS scores, to demonstrate its autoregressive capabilities, in Fig. 11 we provide certain examples, where the model is tasked to predict multiple future scores, based on its previous predictions. We observe that the model is capable of capturing the underlying progression patterns, as the red dots (model’s predictions) generally follow the blue line (ground truth scores). Additionally, PDualNet respects patient-specific history and characteristics, generating smooth and realistic future predictions, without overshooting or undershooting compared to the expected values. This stable, and fully autoregressive performance, is a significant advantage compared to the baseline models, which are constrained to single-step predictions, and can be very beneficial for clinical decision-making, as it gives insight to clinicians regarding the disease’s future course. Furthermore, the model’s subtype predictions appear to reflect the patient’s current and future disease trajectory: clearer patterns of progression tend to yield more confident predictions for a specific subtype (see Fig. 11f), while more ambiguous patterns result in a more evenly distributed set of probabilities across multiple subtypes (see Fig. 11b).

## Conclusions and future work

In this work, a novel end-to-end deep learning framework, PDualNet, is proposed to address the dual-problem of Parkinson’s Disease progression subtype classification and future MDS-UPDRS score prediction. The model exploits the longitudinal nature of the data on two levels: first, by encoding the single visit information into a latent representation (*SiVE*), and second, by identifying the hidden temporal patterns across all provided visits, through a Transformer-based architecture. This structure, combined with the three-phase training procedure, allows the model to learn a shared representation (*DiSE*), which essentially encodes the disease state in terms of progression, and thus being able to address both tasks simultaneously.

Furthermore, the model is trained on a large, broadly representative cohort, the PPMI, and evaluated on both an internal test and an external validation set, derived from the PDBP study. In addition to that, to benchmark performance, we compared PDualNet with three baseline models that are often used in the field to address the same tasks. The results demonstrated the model’s superior performance on both cohorts, highlighting its robustness and generalization capabilities. It is also worth mentioning that, compared to the baselines, PDualNet was able to achieve the highest accuracy, F1 score and lowest MSE for the regression task, all within a single-unified framework. This is a significant advantage over the baseline models, which are constrained to single-step predictions, and specific input sequence lengths. While PDualNet contains more parameters than its counterparts, it is important to highlight that baseline models were trained separately for each of the four input sequence lengths, resulting in comparable or even higher overall complexity and training effort when considering the total number of the required trained models. This further supports the efficiency and flexibility of PDualNet as a general-purpose forecasting framework.

Another limitation is that, although two large datasets were used, the study may not fully capture the diversity encountered in real-world clinical practice. The inevitable data heterogeneity (different quality or lack of data across different institutions) and/or possible inherent biases in training data may cause errors in predictions for underrepresented groups. Therefore, deeper analysis, including a wide spectrum of individual cases, is warranted before clinical deployment can be considered. Additionally, the use of latent representations of data leads inevitably to limited interpretability. Tools such as the SHAP values do address this limitation to a great extent by providing valuable insights regarding the model’s decision-making, as discussed above; however, they do not reflect causation. External validation and targeted causal and bias assessments are required in applications where interpretability is key.

In terms of future work, it would be interesting to integrate additional data sources, and modalities, such as wearable sensor data (e.g., accelerometer), imaging data (e.g. MRI, DaTscan) or genetic information, to further enhance the model’s generalization capabilities and deepen its disease encoding abilities. Multimodal fusion could help the model capture complementary information and disease signals that could lead to improved performance in downstream tasks. Exploring modality-specific encoders or cross-modal attention mechanisms that would enable the transformer to learn richer, clinically meaningful representations of Parkinson’s disease heterogeneity. This could also strengthen the model’s explainability. For instance, the integration of molecular biomarker or neuroimaging data (e.g., DAT-SPECT or MRI-derived features), could unveil stronger subtype-biomarker correlations, enhancing the clinical interpretability and diagnostic relevance of PDualNet. Another promising direction is to explore stacking-based ensemble learning models for combining results from different architectures. As recent studies^52,53^ have shown, such ensemble frameworks can enhance diagnostic accuracy and generalization. Similar models could be employed to fuse the predictions of PDualNet with complementary information from additional modality-specific modules (e.g., imaging, genetic, or sensor data), thereby leveraging their joint strengths to achieve more robust and clinically meaningful decision support. In the context of our work, similar models can be applied to fuse PDualNet results with data from modules processing additional modalities to combine their complementary strengths towards improved clinical decision support. Furthermore, extensions of PDualNet can be proposed by employing multi-modal federated learning for training models without direct access to the raw data held by different clients, thus facilitating clinical deployment and addressing important data privacy and security concerns. Another observation arising from the model’s generic structure is that the encoder component or the learned *DiSE* embeddings alone, can be employed independently in downstream tasks related to the disease (e.g. for predicting longitudinal trajectories of additional clinical scales such as MoCA, ESS, Schwab and England ADL score, etc.).

## Data Availability

Data used in the preparation of this article were obtained from the Accelerating Medicine Partnership$^\circledR$ (AMP$^\circledR$) Parkinson’s Disease (AMP PD) Knowledge Platform. For up-to-date information on the study, visit https://www.amp-pd.org. “The AMP$^\circledR$ PD program is a public-private partnership managed by the Foundation for the National Institutes of Health and funded by the National Institute of Neurological Disorders and Stroke (NINDS) in partnership with the Aligning Science Across Parkinson’s (ASAP) initiative; Celgene Corporation, a subsidiary of Bristol-Myers Squibb Company; GlaxoSmithKline plc (GSK); The Michael J. Fox Foundation for Parkinson’s Research ; Pfizer Inc.; Sanofi US Services Inc.; and Verily Life Sciences. “ACCELERATING MEDICINES PARTNERSHIP and AMP are registered service marks of the U.S. Department of Health and Human Services. More specifically, we obtained the clinical data corresponding to the MJFF Parkinson’s Progression Markers Initiative (PPMI) and the National Institute of Neurological Disorders and Stroke (NINDS) Parkinson’s Disease Biomarkers Program (PDBP) (AMP-PD, 2023 releases, v4 release). PPMI–a public-private partnership–is funded by the Michael J. Fox Foundation for Parkinson’s Research and funding partners, including 4D Pharma, Abbvie, AcureX, Allergan,Amathus Therapeutics, Aligning Science Across Parkinson’s, AskBio, Avid Radiopharmaceuticals, BIAL, BioArctic, Biogen, Biohaven, BioLegend, BlueRock Therapeutics, Bristol-Myers Squibb, Calico Labs, Capsida Biotherapeutics, Celgene, Cerevel Therapeutics, Coave Therapeutics, DaCapo Brainscience, Denali, Edmond J. Safra Foundation, Eli Lilly, Gain Therapeutics, GE HealthCare, Genentech, GlaxoSmithKline plc (GSK), Golub Capital, Handl Therapeutics, Insitro, Janssen Neuroscience, Jazz Pharmaceuticals, Lundbeck, Merck, Meso Scale Discovery, Mission Therapeutics, Neurocrine Biosciences, Neuropore, Pfizer, Piramal, Prevail Therapeutics, Roche, Sanofi, Servier, Sun Pharma Advanced Research Company, Takeda, Teva, UCB, Vanqua Bio, Verily, Voyager Therapeutics, the Weston Family Foundation and Yumanity Therapeutics. The PPMI investigators have not participated in reviewing the data analysis or content of the manuscript. For up-to-date information on the study, visit https://www.ppmi-info.org. PDBP is supported by the National Institute of Neurological Disorders and Stroke at the National Institutes of Health. Investigators include: Roger Albin,Roy Alcalay, Alberto Ascherio, Thomas Beach, Sarah Berman,Bradley Boeve, F. DuBois Bowman, Shu Chen, Alice Chen-Plotkin, William Dauer, Ted Dawson, Paula Desplats, Richard Dewey, Ray Dorsey, Jori Fleisher, Kirk Frey, Douglas Galasko, James Galvin, Dwight German, Lawrence Honig, Xuemei Huang, David Irwin, Kejal Kantarci, Anumantha Kanthasamy, Daniel Kaufer, James Leverenz, Carol Lippa, Irene Litvan, Oscar Lopez, Jian Ma, Lara Mangravite, Karen Marder, Laurie Orzelius, Vladislav Petyuk, Judith Potashkin, Liana Rosenthal, Rachel Saunders- Pullman, Clemens Scherzer, Michael Schwarzschild, Tanya Simuni, Andrew Singleton, David Standaert, Debby Tsuang, David Vaillancourt, David Walt, Andrew West, Cyrus Zabetian, Jing Zhang, and Wenquan Zou. The PDBP Investigators have not participated in reviewing the data analysis or content of the manuscript. For up-to-date information on the study, visit: https://pdbp.ninds.nih.gov.
